# Diabetes is often overlooked after a myocardial infarction

**Published:** 2016

At least 10% of people who have a myocardial infarction (MI) may also have undiagnosed diabetes. Yet many doctors fail to look for diabetes in these patients, a recent study has found.

Dr Suzanne Arnold, assistant professor at Saint Luke’s Mid-America Heart Institute and the University of Missouri in Kansas City, and her team studied data from 2 854 patients who experienced an MI and had never been diagnosed with type 2 diabetes. The study tested the patients’ HbA_1c_ levels.

It revealed that doctors often failed to recognise and begin treating diabetes in patients who have experienced MIs with no prior history of diabetes, even when the patient testedpositive for diabetes. The researchers found that 287 or 10.1% of the patients who experienced MIs tested positive for diabetes. Out of the 287 patients who tested positive for diabetes, less than one-third received education or medication when discharged from hospital.

According to the results, doctors failed to recognise diabetes in 198 or 69% of the previously undiagnosed patients. The researchers noted that when a patient’s HbA_1c_ test results were checked while they were being treated for their MI, there was a 17-fold greater chance that the diabetes would be diagnosed.

In a press release, Dr Arnold stated, ‘Diagnosing diabetes in patients who have had a heart attack is important because of the role diabetes plays in heart disease. By recognising and treating diabetes early, we may be able to prevent additional cardiovascular complications through diet, weight loss and lifestyle changes, in addition to taking medications. Another important reason to diagnose diabetes at the time of heart attack is that it can guide the treatments for the patient’s coronary artery disease.’

According to Dr Arnold and her team, two in three patients with diabetes die from heart-related conditions. Patients with diabetes experience a significantly higher risk for MI. The authors concluded that people who have an MI should ask for a diabetes test if they present with other risk factors such as being overweight, having high blood pressure or a family history of diabetes.

This study was presented on 3 June at the American Heart Association’s Quality of Care and Outcomes Research Scientific Sessions 2014.
